# Mapping Tumor–Stroma–ECM Interactions in Spatially Advanced 3D Models of Pancreatic Cancer

**DOI:** 10.1021/acsami.5c02296

**Published:** 2025-03-07

**Authors:** Anna-Dimitra Kataki, Priyanka G. Gupta, Umber Cheema, Andrew Nisbet, Yaohe Wang, Hemant M. Kocher, Pedro A. Pérez-Mancera, Eirini G. Velliou

**Affiliations:** † Centre for 3D models of Health and Disease, Division of Surgery and Interventional Science, University College London, London W1W 7TY, U.K.; ‡ School of Life and Health Sciences, Whitelands College, University of Roehampton, London SW15 4JD, U.K.; § Department of Medical Physics and Biomedical Engineering, University College London, London WC1E 6BT, U.K.; ∥ Centre for Cancer Biomarkers and Biotherapeutics, Barts Cancer Institute, Queen Mary University of London, London EC1M 6BQ, U.K.; ⊥ Centre for Tumour Biology and Experimental Cancer Medicine, Barts Cancer Institute, Queen Mary University of London, London EC1M 6BQ, U.K.; # Department of Molecular and Clinical Cancer Medicine, University of Liverpool, Liverpool L69 3GE, U.K.

**Keywords:** pancreatic cancer, extracellular matrix, 3D models, fibrosis, multicellular models, tumor microenvironment, cancer models, stellate cells

## Abstract

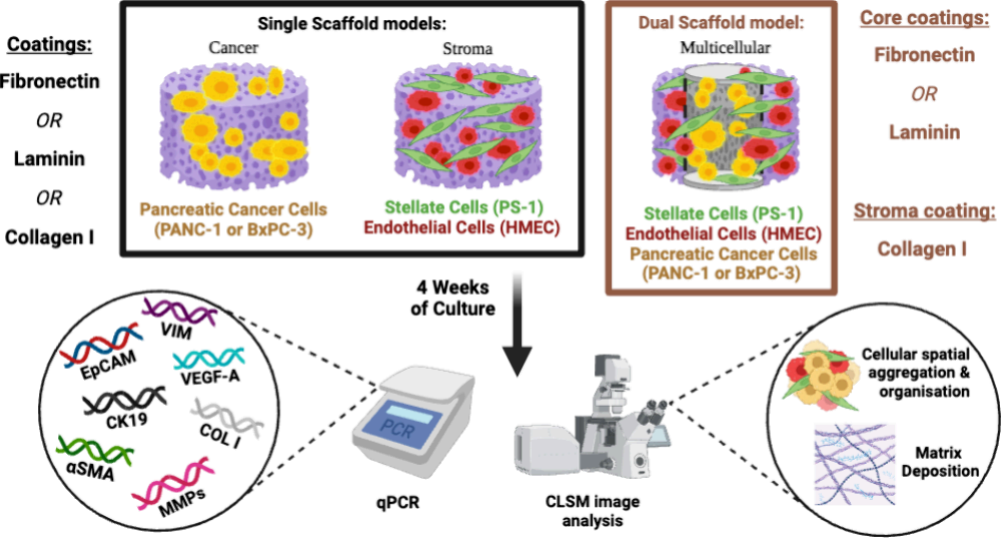

Bioengineering-based *in vitro* tumor models are increasingly important as tools for studying disease progression and therapy response for many cancers, including the deadly pancreatic ductal adenocarcinoma (PDAC) that exhibits a tumor/tissue microenvironment of high cellular/biochemical complexity. Therefore, it is crucial for *in vitro* models to capture that complexity and to enable investigation of the interplay between cancer cells and factors such as extracellular matrix (ECM) proteins or stroma cells. Using polyurethane (PU) scaffolds, we performed a systematic study on how different ECM protein scaffold coatings impact the long-term cell evolution in scaffolds containing only cancer or only stroma cells (activated stellate and endothelial cells). To investigate potential further changes in those biomarkers due to cancer–stroma interactions, we mapped their expression in dual/zonal scaffolds consisting of a cancer core and a stroma periphery, spatially mimicking the fibrotic/desmoplastic reaction in PDAC. In our single scaffolds, we observed that the protein coating affected the cancer cell spatial aggregation, matrix deposition, and biomarker upregulation in a cell-line-dependent manner. In single stroma scaffolds, different levels of fibrosis/desmoplasia in terms of ECM composition/quantity were generated depending on the ECM coating. When studying the evolution of cancer and stroma cells in our dual/zonal model, biomarkers linked to cell aggressiveness/invasiveness were further upregulated by both cancer and stroma cells as compared to single scaffold models. Collectively, our study advances the understanding of how different ECM proteins impact the long-term cell evolution in PU scaffolds. Our findings show that within our bioengineered models, we can stimulate the cells of the PDAC microenvironment to develop different levels of aggressiveness/invasiveness, as well as different levels of fibrosis. Furthermore, we highlight the importance of considering spatial complexity to map cell invasion. Our work contributes to the design of *in vitro* models with variable, yet biomimetic, tissue-like properties for studying the tumor microenvironment’s role in cancer progression.

## 1 Introduction

Termed as a “silent killer,” pancreatic ductal adenocarcinoma (PDAC), a malignancy of the pancreas, exhibits a devastating 5-year survival rate of only ∼13% with minimal improvement observed over the past decade.^1^ A key factor contributing to these disheartening outcomes is the significant resistance of PDAC to conventional treatment modalities, including chemotherapy and radiotherapy.^2,3^ This resistance and aggressive disease progression is partly attributed to the PDAC’s intricate tumor microenvironment (TME), which comprises a diverse array of cellular, biochemical, biophysical, and structural elements, which interact in complex ways, leading to the progression of the disease and driving the response to therapies.^4^ Notably, during disease progression, activated stellate cells within the pancreas contribute to excessive extracellular matrix (ECM) protein deposition, culminating in the formation of desmoplasia/fibrosis around the tumor, which is a hallmark of the disease and a major driver of treatment resistance.^5−7^

The ECM plays a pivotal role in shaping the TME of PDAC. The ECM of the PDAC TME is composed of a diverse array of proteins, such as collagens, fibronectin, laminin, and proteoglycans.^3,8−10^ The ECM functions as the structural framework for the tissue’s cells to sit in, adhere, and proliferate while providing mechanical and physiochemical cues through cell–matrix interactions that influence cellular behavior.^3,8−10^ For example, increased deposition of collagen, laminin, and fibronectin has been associated with enhanced PDAC progression and poor patient outcomes.^11−14^ Additionally, the presence of certain ECM proteins has been linked to reduced therapeutic response and patient survival.^11−13,15,16^ For example, Chen et al., in a study involving 37 patients, showed that metastatic patient samples overexpressed laminin, which was associated with poor prognosis.^12^ In an *in vitro* study, Usman et al. investigated the impact of ECM proteins (fibronectin, collagen I, laminin, and vitronectin) on the cell spreading and proliferation of various PDAC cell lines in a 2D culture for 6 days and showed that fibronectin and vitronectin affected the spreading/proliferation in a cell-line dependent manner.^16^ Therefore, understanding the dynamic interplay between ECM composition, ECM remodeling, and cellular behavior is critical for developing novel therapeutic strategies to target the TME in PDAC.^9,10,14,17^

PDAC research has traditionally relied on either 2D *in vitro* systems or *in vivo* animal models. While 2D *in vitro* systems (tissue culture flasks or plates) offer ease of use, reproducibility, and cost-effectiveness, they lack the ability to effectively replicate the *in vivo* tumor tissue characteristics, such as three-dimensionality, structure, stiffness, cellular spatial organization, cell–cell and cell–ECM interactions, as well as *in vivo*-like biochemical cues and metabolic gradients.^18−25^ In contrast, animal models provide a more realistic representation of *in vivo* tissue conditions; however, they are associated with limited reproducibility, ethical considerations, and high costs.^5,19,20,26−28^ All the above have led to an urgent need for investigating alternative *in vitro* models to bridge the gap between 2D cultures and *in vivo* models, to enable a more biomimetic approach toward PDAC’s TME, and to facilitate a better understanding of the interplay between the TME's various components.^5,18−20,26−38^

In recent years, three-dimensional (3D) *in vitro* models have emerged as valuable tools for studying the complex interplay between tumor cells and their microenvironment.^5,18,20,32,34,35,37,39−45^ Commonly used 3D PDAC models include (i) spheroids/organoids, (ii) hydrogels, and (iii) polymer-based scaffolds.

Spheroids/organoids are self-assembled cellular structures/cell aggregates (either monocellular or multicellular) composed of cell lines or patient-derived tissues that partly mimic the three-dimensional spatial organization and, in the case of multicellular spheroids, the cellular heterogeneity observed *in vivo*. These systems allow for the investigation of cell–cell and cell–ECM interactions on tumor growth and response to therapy.^25,46−65^ For example, Monteiro et al. fabricated monotypic (cancer cells only) and heterotypic (cancer and stroma cells) spheroids of PANC-1 and human pancreatic CAFs that were cultured for up to 14 days. It was observed that unlike monocellular spheroids, the multicellular spheroids did not develop a clear necrotic core, likely due to stromal cell paracrine signaling that supported cancer cell survival. Additionally, these 3D models remained structurally compact and viable for up to 14 days. Drug resistance and the expression of invasion-related factors like CXCL12 and MMP9 were higher in the multicellular spheroids, indicating enhanced drug resistance potential.^31^ Liu et al. cultured monocellular pancreatic cancer spheroids (PANC-1) or multicellular spheroids (PANC-1 in co-culture with murine pancreatic stellate cells (mPSCs)) to test tumor-specific chemosensitivities with the drugs gemcitabine, paclitaxel, SN38, and pitavastatin. They observed that co-culture with stellate cells shifted the cancer cells toward a more aggressive molecular subtype as observed by significant upregulation of the biomarkers Cxcl1, Il6, Il1r1, Acta2, and Ctgf at the mRNA level. In their multicellular spheroids, murine stellate cells were activated, becoming myofibroblastic (myCAF) and inflammatory CAFs (iCAFs). In addition, the multicellular spheroids showed reduced drug sensitivity of the cancer cells.^66^ Norberg et al. developed a multicellular spheroid model of PDAC, with PANC-1 or HPAFII cancer cells and human pancreatic stellate cells (hPSCs). They saw that, in multicellular spheroids, the presence of the hPSCs completely suppressed the expression of the epithelial marker E-cadherin in PANC-1 cells, possibly promoting a more mesenchymal phenotype, while HPAFII cells strongly expressed E-cadherin regardless of the presence of hPSCs.^60^

Hydrogels are 3D networks of cross-linked polymer chains (of natural or synthetic polymers) capable of retaining large amounts of water. By tuning the composition and mechanical properties of hydrogels, researchers can recreate and accurately control various aspects of the tumor TME, such as the structure/internal architecture and porosity, the stiffness as well as biochemical cues (by inclusion of ECM proteins or peptides), enabling tissue–ECM mimicry.^25,33,34,67−80^ For example, Osuna de la Peña et al. developed a hydrogel system using self-assembling peptide amphiphiles and a mixture of PDAC-relevant ECM components (laminin, hyaluronan, fibronectin, collagen I) to recapitulate the PDAC TME. They encapsulated patient-derived PDAC cells, pancreatic stellate cells, and macrophages within these hydrogels and maintained long-term cultures for up to 21 days. Notably, the encapsulated cells preserved their native morphology, ductal topology, and gene expression profiles akin to *in vivo* and *ex vivo* PDAC tumors, demonstrating the physiological relevance of this 3D model for studying the complex PDAC microenvironment.^33^ Below et al. developed a 3D triculture PEG hydrogel cross-linked with peptides that were sensitive to matrix metalloproteinases (MMPs) for support of 3D *in vitro* growth of pancreatic organoids.^34^ The model robustly mimicked the physiological stiffness and ECM of PDAC, replicated cell–ECM interactions, and supported *in vitro* organoid growth. Usman et al. developed a PEG-based hydrogel system with incorporation of either fibronectin or laminin and seeded with MiaPaCa-2 pancreatic cancer cells. They did not observe any difference in morphology and proliferation rate between the different proteins, unlike 2D cultures on protein-coated plates.^16^

Polymeric scaffolds are versatile 3D networks made from synthetic polymers designed to mimic various aspects of the TME. These scaffolds allow precise control over the mechanical properties and internal architecture, similar to hydrogels, enabling the study of tissue stiffness and structure. Additionally, they can be functionalized with ECM proteins to replicate specific tissue characteristics and facilitate the examination of ECM–cell interactions, providing accurate models for cancer research.^22,23,29,35,37,38,81,82^ For example, Ricci et al. developed three biocompatible polymeric scaffolds, which were seeded with human primary PDAC cells and cultured for up to 9 days. These scaffolds were composed of a poly(vinyl alcohol)/gelatin (PVA/G) blend and a poly(ethylene oxide terephthalate)/poly(butylene terephthalate) (PEOT/PBT) copolymer, each featuring distinct internal architectures, such as sponge-like structures and nanofiber meshes. The study focused on evaluating cell morphology, differentiation, and spatial organization within the different scaffolds, as well as analyzing the expression of MMPs, enzymes crucial for ECM remodeling during cancer progression. It was shown that the sponge-like porous scaffolds promoted cellular aggregation and increased MMP activity, closely resembling the native PDAC tissue architecture, compared to the fibrous meshes.^81^ We have previously developed both monocellular and zonal multicellular, polyurethane (PU) scaffold-based models for PDAC, which can be surface-modified with proteins for ECM mimicry, wherein we have shown growth and proliferation for multiple cell types (cancer and stroma), desmoplasia mimicry, and feasibility of therapeutic assessment (chemotherapy, radiotherapy, and chemoradiotherapy).^27,35−38^ More specifically, our multicellular PDAC model is comprised of (i) a fibronectin-coated inner cylinder/core compartment, in which pancreatic cancer cells are seeded (PANC-1 cells), surrounded by (ii) a collagen I-coated outer cuboid, in which activated stellate cells (PS-1) and endothelial cells (HMECs), i.e., the stroma, are seeded. Such architectural configuration spatially mimics the *in vivo* PDAC TME. Our work has conclusively shown the impact of stromal cells along with zonal/spatial segregation of cancer and stroma compartments on cellular growth, distribution, phenotype, and response to chemotherapeutic treatment.^36,37,83^

It is evident from the above studies that biomaterial-based 3D models that include ECM proteins have a great potential for *in vitro* cancer studies of PDAC and for therapy screening. However, there is a need for more research on ECM–cell interactions in 3D models to shed light on their role in *in vitro* cell growth and therapeutic response. With the exception of the work of Usman et al., who systematically compared the growth of pancreatic cancer cells in PEG hydrogels cross-linked with various ECM proteins, to the best of our knowledge to date, there is no study systematically varying and comparing the impact of ECM proteins of a specific 3D model on PDAC cell evolution *in vitro*.^16^ This is an important aspect, especially since, as previously mentioned, there is clinical evidence that the presence/abundance of certain proteins in the matrix of pancreatic cancer can affect the progression of the disease and the therapy resistance.^9,10,12,84^

**Figure 1:**
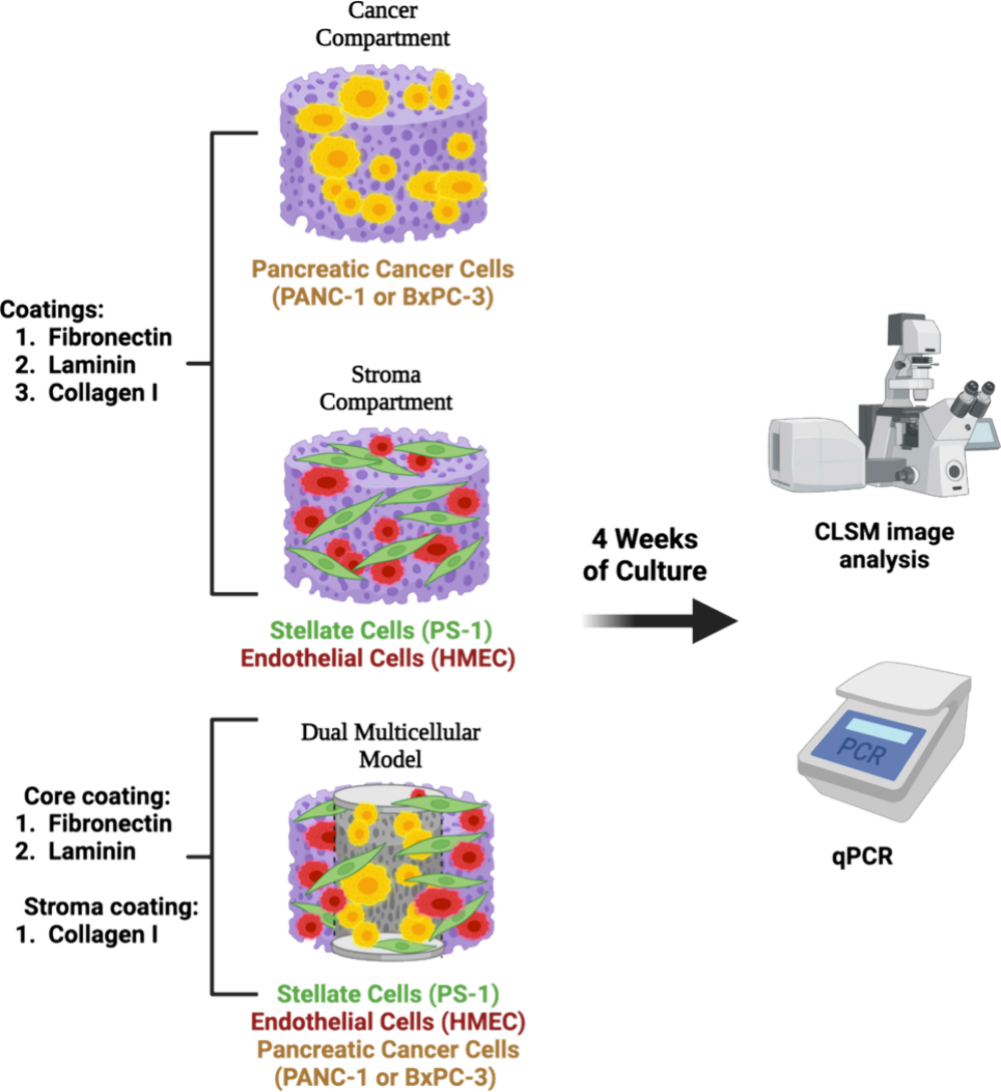
Schematic representation of the experimental design for tailoring the ECM complexity on PU scaffold-based monocellular and multicellular PU-based PDAC models (Created with Biorender.com).

In the current study, we investigate the effect of changes to ECM protein coatings in our PU scaffolds on the cellular evolution for both PDAC and stroma cells (Figure 1). More specifically, we performed a systematic comparative study of PDAC and the stroma cellular evolution (spatial distribution, growth, ECM deposition) and biomarker profiling (biomarkers linked to a more invasive cell phenotype and/or therapy resistance) in our scaffolds for 3 different ECM proteins, namely, fibronectin (FN), collagen I (COL I), and laminin (LAM), in (i) a single scaffold cancer model (monocellular, consisting of cancer cells only), (ii) a single scaffold stroma model (multicellular consisting of a co-culture of activated stellate cells and endothelial cells), (iii) a multicellular (tri-culture) zonal/dual scaffold model consisting of a central cancer compartment, surrounded by an external stroma compartment, the latter spatially mimicking the desmoplastic reaction in PDAC (Figure 1). Our work sheds light on the impact that different ECM proteins of the scaffold/model can have on the evolution of cancer and stroma cells of the PDAC tissue environment.

## 2 Materials and Methods

### 2.1 Polymer Scaffold Preparation and Surface Modification

Polyurethane (PU) scaffolds were fabricated via the thermal induced phase separation (TIPS) method as we have previously described.^27,35,38,85^ The scaffolds were then cut at appropriate sizes (see Sections 2.3.1 and 2.3.2). Thereafter, sterilisation took place by exposure of the scaffolds to 70% ethanol (3 h) and UV ray (1 h). Surface modification of the resulting scaffolds was carried out with ECM proteins for ECM biomimicry.^36,37^ More specifically, scaffolds were surface modified with fibronectin (FN), laminin (LAM), or collagen I (COL I) depending on the experimental condition (Figure 1), see also sections Sections 2.3.1 and 2.3.2.

### 2.2 2D Cell Culture

The *human pancreatic adenocarcinoma cell line PANC-1* (ATCC, U.K.) was expanded in Dulbecco’s modified Eagle’s medium (DMEM) with high glucose (Sigma-Aldrich, Merck, U.K.), supplemented with 10% fetal bovine serum (FBS, Fisher Scientific, U.K.), 1% penicillin/streptomycin (Fisher Scientific, U.K.), and 2 mM l-glutamine (Sigma-Aldrich, Merck, U.K.) in a humidified incubator at 37 °C with 5% CO_2_.

The *human pancreatic adenocarcinoma cell line BxPC-3* (ATCC, U.K.) was expanded in RPMI 1640 (ATCC modification) medium (GIBCO, Thermo Fisher, U.K.) supplemented with 10% FBS (Fisher Scientific, U.K.) and 1% penicillin/streptomycin (Fisher Scientific, U.K.) in a humidified incubator at 37 °C with 5% CO_2_.

The above two cell lines were selected to represent different stages of the PDAC disease, namely, PANC-1 constitutes a poorly differentiated and highly metastatic cell line that exhibits resistance to gemcitabine treatment.^86,87^ BxPC-3 is a nonmetastatic and moderately-to-poorly differentiated cancer cell line that is known to exhibit sensitivity to gemcitabine treatment.^86,87^

The *human microvascular endothelial cell (HMEC) line CRL-3243* (ATCC, U.K.) was expanded in MCDB 131 medium (GIBCO, Thermo Fisher, U.K.) supplemented with 10% FBS, 1% penicillin/streptomycin, 2 mM l-glutamine, 10 ng/mL epidermal growth factor (Sigma-Aldrich, Merck, U.K.), and 1 μg/mL hydrocortisone (Sigma-Aldrich, Merck, U.K.) in a humidified incubator at 37 °C with 5% CO_2_. This cell line was chosen to represent/mimic the endothelial population of the tumor tissue.^37^

The *immortalized human pancreatic stellate cell line PS-1*(45,88) was expanded in DMEM (SIGMA-Aldrich, Merck, U.K.) supplemented with 10% FBS (Fisher Scientific, U.K.), 1% penicillin/streptomycin (Fisher Scientific, U.K.), and 2 mM l-glutamine (Sigma-Aldrich, Merck, U.K.) in a humidified incubator at 37 °C with 5% CO_2_.

All cells were passaged regularly on reaching 80–90% confluency with TrypLE (GIBCO, Thermo Fisher, U.K.) until the appropriate cell densities were reached.

### 2.3 3D Pancreatic Tumor Models

#### 2.3.1 Single Scaffold 3D Models (Cancer or Stroma Compartment Model)

We initially investigated the impact of different protein coatings on either PDAC cells (PANC-1 or BxPC-3 PDAC cells) or cells of the stroma (PS-1 cells and HMECs seeded in the PU scaffolds in a 1:1 seeding ratio; Figure 1). For these experiments, cells were seeded in single PU scaffolds (5 × 5 × 5 mm^3^) coated with either fibronectin (FN), collagen I (COL I), or laminin (LAM). The cell density used was 0.5 × 10^6^ cells/scaffold/cell type, as we have previously reported.^27,35−38^ Thereafter, the scaffolds were placed in 24-well plates and cultured for 28 days (4 weeks) in a humidified incubator at 37 °C with 5% CO_2_.^27,35−38^

#### 2.3.2 Dual Scaffold Multicellular 3D Models

Based on the results obtained in the single scaffold models, selected protein coatings were used to coat either the cancer or the stroma compartment of our multicellular model (Figure 1) in order to investigate potential further changes in key biomarkers (linked to a more invasive/aggressive cell phenotype and/or to treatment resistance) in both the cancer and the stroma cell populations as a result of their interactions (dual multicellular 3D model). As we have previously described, our multicellular model is a dual (zonal) scaffold-based model, which recapitulates the spatial architecture of the PDAC TME.^36,37,83^

Two separate PU zones (a hollow cuboid/external ring with dimensions of 7 × 7 × 5 mm^3^ and a solid inner cylinder/core of diameter 3 mm and height of 5 mm) were fabricated. The outer cuboid/external ring was coated with COL I and seeded with stroma cells, while the inner cylinder was coated with FN or LAM and seeded with PDAC cancer cells (Figure 1). More specifically, 0.25 × 10^6^ PANC-1 or BxPC-3 cancer cells were seeded in the inner FN- or LAM-coated cancer compartment, respectively (resuspended in 10 μL of media), and cultured for 7 days. This cell number was selected, as per our previous studies,^36,37^ to ensure the same cell spatial density as the single scaffold configuration. After 7 days, PS-1 stellate cells and HMEC cells were added to the outer stroma compartment at a seeding density of 0.5 × 10^6^ cells/cell type and then plugged together with the inner cylinder to assemble the complete hybrid zonal model. Thereafter, the tri-culture was monitored for an additional 21 days (total culturing period was 4 weeks). The difference in seeding times between the cancer core and the stroma external ring was selected based on our previous studies to ensure optimal growth of cell types by week 4 of culture and to avoid *in vitro* aging of the stroma.^36,37^ The cell ratio was PANC-1/PS-1/HMEC = 1:2:2 and BxPC-3/PS-1/HMEC = 1:2:2 at the time of seeding.^36,37^

### 2.4 Viability Mapping in the 3D Scaffolds

To visualize the spatial distribution of live and dead cells, scaffolds were snap frozen, at week 4 of culture, in liquid nitrogen for 15 min and then preserved at −80 °C.^27,35−38^ For live/dead cell analysis, the Live/Dead Viability/Cytotoxicity kit was used (Molecular Probes, Thermo Scientific, U.K.). Prior to analysis, scaffolds were sectioned, stained with 2 μM Calcein-AM (4 mM stock) and 4 μM ethidium homodimer (2 mM stock), and incubated at 37 °C for 1 h. The solution was then removed, and samples were washed twice in PBS, followed by imaging using a Zeiss 880 inverted confocal microscope (Zeiss, U.K.).^36,37^

### 2.5 Immunofluorescence Assays

*In situ* immunofluorescence (IF) staining of the scaffolds was carried out for spatial determination of (i) the different cell types with von Willebrand factor (VWF) or CD31 (HMEC), αSMA (PS-1), and pan-Cytokeratin (PANC-1) and (ii) cellular deposition of ECM proteins (FN, COL I, LAM). As per Section 2.4, scaffolds were snap frozen at specific time points and further processed. More specifically, scaffolds were sectioned and fixed for 2–4 h in 4% w/v paraformaldehyde (Sigma-Aldrich, Merck, U.K.). Sections were then permeabilized for 2 h with 0.1% Triton -X solution (SIGMA-Aldrich, Merck, U.K.). This was followed by a series of sequential blocking with either 10% donkey serum or 10% rabbit serum solution, and overnight staining with primary antibodies (Supporting Information Table S1) and secondary antibodies (Supporting Information Table S1) was carried out. All samples were costained with DAPI (Thermo Fisher, U.K.). Each step employed a solvent containing 1% w/v bovine serum albumin (Sigma-Aldrich, Merck, U.K.) and 0.5% v/v Tween-20 (Promega, U.K.).^36^

### 2.6 Confocal Laser Scanning Microscopy (CLSM) Imaging

Immunofluorescent samples were imaged with a Zeiss 880 inverted confocal microscope (Zeiss, U.K.) and processed with Zen Black software using the following lasers and filters: (i) 405 nm (for DAPI), (ii) 488 nm (for Alexa Fluor 488, Dylight 488), (iii) 561 nm (for Alexa Fluor 555, Dylight 550), and (iv) 643 nm (for Alexa Fluor 647, Dylight 650) for 2 sequential scans. Confocal images were captured using a 10× dry objective, with a 512 × 512 pixel resolution and a 15–30 μm Z-stack distance, as previously described. Multiple scaffolds (at least three) and multiple sections (>3) per scaffold were imaged to ensure reproducibility. Representative images are presented in this manuscript.^27,36−38^

### 2.7 mRNA Extraction, cDNA Synthesis, and qPCR (Quantitative Polymerase Chain Reaction) Analysis

Total RNA from all 3D models under study was extracted at 4 weeks of culture using the RNeasy mini kit (Qiagen, U.K.) as per manufacturer’s instructions and stored at −80 °C. More specifically, the single scaffold model was used as a whole for mRNA extraction, whereas for the dual scaffold model, the inner cancer compartment and outer stroma compartment were separated, and mRNA extraction (and thus cDNA synthesis and qPCR analysis) was performed separately for each scaffold compartment.

The total RNA obtained was quantified and assessed for integrity using a NanoDrop. cDNA synthesis was carried out using the RevertAid H Minus Firsti Strand cDNA Synthesis Kit (Thermo Scientific, U.K.) on a T100 Thermal Cycler (Bio-Rad, Watford, U.K.). cDNA was stored at −20 °C. Minimum Information for Publication of Quantitative Real-Time PCR Experiments (MIQE) guidelines was followed during designing of primer pairs^89^ (Supporting Information Table S2). The annealing temperature was set to 60 °C, and the primer pairs were obtained from Eurofins Genomics (Ebersberg, Germany). iTaq Universal SYBR Green Supermix was used to amplify the target gene in 10 μL reactions composed of 20 ng of sample and 0.2 μM primer concentration. The reaction ran for 40 cycles on the CFX96 Touch System (both from Bio-Rad, Watford, U.K.). Each sample was tested in triplicate. The ΔCq^90^ was used to analyze the relative gene expression normalized to the reference gene β-Actin (ACTB) for PANC-1 and BxPC-3 cancer cells and glyceraldehyde 3-phosphate dehydrogenase *(GAPDH) for PS-1 and HMEC cells. The following biomarkers were assessed for the cancer compartment (PANC-1 or BxPC-3) for all scaffold configurations under study (Figure 1): (i) Epithelial markers epithelial cell-adhesion molecule (EpCAM) and cytokeratin-19 (CK19), (ii) mesenchymal marker vimentin (VIM), (iii) MMPs that are linked/associated with matrix remodeling and cell invasiveness (MMP2 and MMP9), and (iv) vascular endothelial growth factor A (VEGFA).^73,91−103^ The following markers were assessed for the stroma compartment (PS-1 and HMEC) for all scaffold configurations under study: (i) the myofibroblastic marker alpha smooth muscle actin (αSMA), (ii) the mesenchymal marker vimentin (VIM), (iii) collagen I (COL I), and (iv) MMPs (MMP2 and MMP9).^3,14,36,97−100^ The selected biomarkers serve critical roles in assessing different aspects of pancreatic cancer behavior. More specifically, EpCAM is widely recognized as a marker of epithelial cells and is often overexpressed in epithelial cancers, and plays a crucial role in cell adhesion, proliferation, and differentiation, making it an important biomarker for assessing the epithelial characteristics of cancer cells in 3D models.^92,93,103^ CK19 is an intermediate filament protein expressed in epithelial tissues, including pancreatic cancer cells. Its presence is indicative of the epithelial origin of cancer cells, and its expression is often associated with the aggressive nature of PDAC, making it a critical marker for evaluating epithelial integrity and tumor progression.^96,101,102^ VIM is a key mesenchymal marker that is also associated with epithelial–mesenchymal transition (EMT), a process that contributes to cancer invasion and metastasis. The expression of VIM in PDAC models is indicative of a mesenchymal, invasive cell phenotype.^73,91,98,101^ MMP2 and MMP9 are enzymes that degrade components of the ECM, facilitating tumors to transition to a more aggressive or metastatic phenotype. Their expression is closely linked to tumor remodeling and the invasive potential of PDAC.^3,94,98−100,104^ VEGFA is a key regulator of angiogenesis, the process by which new blood vessels form from existing ones, and inflammation, which is crucial for tumor growth and survival.^94,95,97^ Finally, COL I is a major component of the ECM and is usually overexpressed in PDAC, contributing to the dense fibrotic stroma characteristic of this cancer. Its expression is linked to the activation of pancreatic stellate cells, which, as previously mentioned, promote tumor growth, invasion, and resistance to therapy.^14,36^

### 2.8 Statistical Analysis

Statistical analysis was performed for at least 3 independent experiments with at least 3 replicates per time-point (*N* ≥ 3, *n* ≥ 3). An unpaired *t* test using the Graph Pad Prism software (version 9.5 for Macintosh) was carried out to find statistically significant differences between data (*p* < 0.05). The error bars in the graphs represent standard error of the mean.

## 3 Results and Discussion

Within this work, we systematically studied the impact of ECM protein coatings of our PU scaffolds on cell growth, spatial distribution, matrix deposition, and upregulation of biomarkers linked to an invasive/aggressive cell phenotype, which is associated with processes such as matrix remodeling, metastasis, and therapy resistance for (i) pancreatic cancer cells (see Section 3.1) and (ii) stroma cells (see Section 3.2). More specifically, as described above, metastatic (PANC-1) and nonmetastatic (BxPC-3) pancreatic cancer cells, as well as stroma cells (activated stellate cells, PS-1 and endothelial cells, HMEC), were cultured in single scaffold configurations, i.e., cancer-only or stroma-only models, coated with the different ECM protein coatings, i.e., FN, COL I, or LAM, for 4 weeks (see also Figure 1). These single scaffold configurations were studied to assess the impact of different ECM protein coatings on cellular growth, spatial distribution, cellular secretion/deposition of key ECM proteins, and mRNA level expression of biomarkers linked to a more aggressive/invasive phenotype of the disease, which are associated with hallmarks like epithelial-to-mesenchymal transition (EMT), matrix modeling, therapy resistance and invasion, and recurring disease. Thereafter, selected ECM coatings for each of the cancer and stroma single scaffold models were employed to assess the further impact of multicellularity on the cellular evolution (as per the monocellular models) using our multicellular dual scaffold model^18,36,37^ (Figure 1).

Overall, our work highlights that the protein coating of the scaffolds can affect cell evolution, leading to microenvironments of different features in terms of desmoplastic/fibrotic levels, as well as invasive potential. Furthermore, we demonstrate that crosstalk between cancer and stroma cells further enhances the upregulation of key biomarkers linked to cell invasiveness/aggressiveness.

### 3.1 3D PDAC Monocellular (Cancer-Only) Models

**Figure 2:**
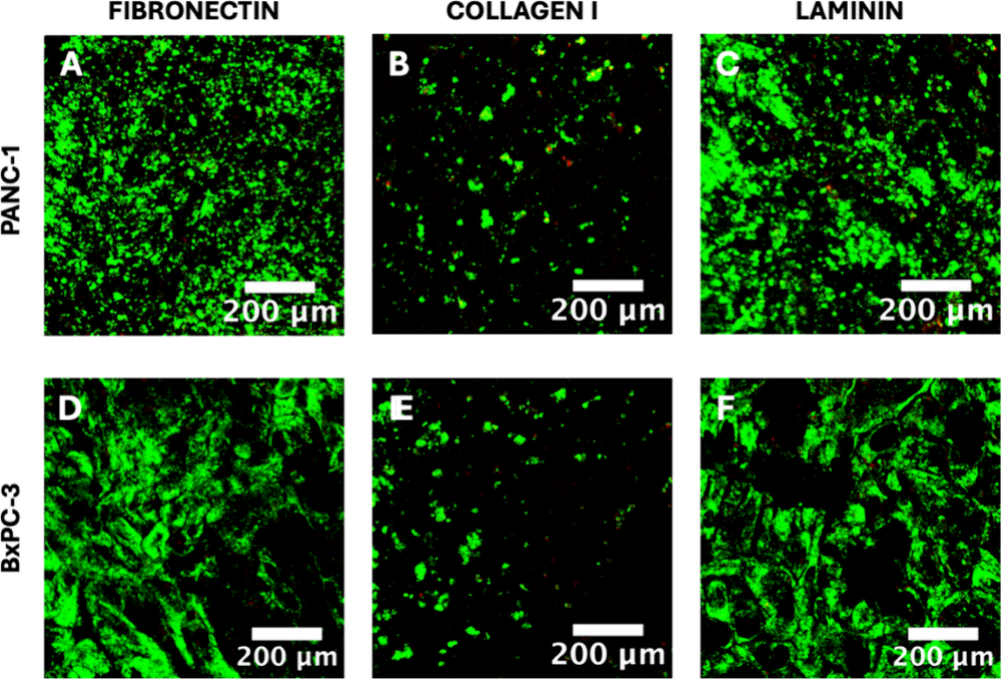
Representative immunofluorescence CLSM images of sections of the single scaffold cancer models for all three ECM protein coatings under study for PANC-1 (panels A–C) or BxPC-3 (panels D–F) pancreatic cancer cells, following live–dead staining at week 4 of culture. Green/red areas signify live/dead cell populations, respectively. Scale bar = 200 μm.

**Figure 3:**
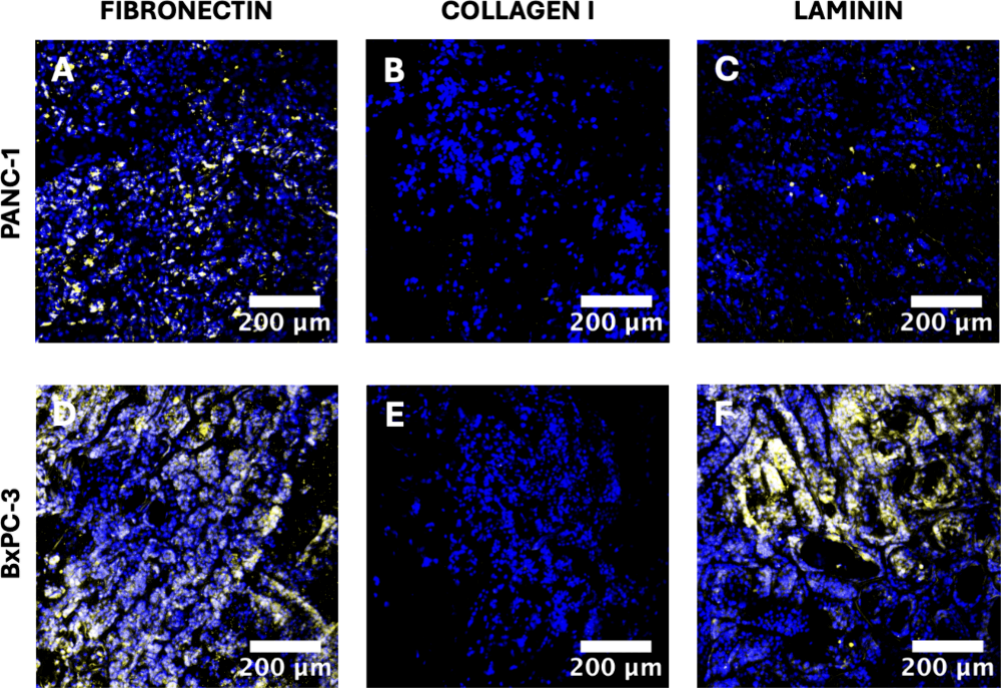
Representative immunofluorescence CLSM images of sections of the single scaffold cancer models for all three ECM protein coatings under study for PANC-1 (panels A–C) or BxPC-3 (panels D–F) pancreatic cancer cells, showing deposition of collagen I (human collagen I in yellow) at week 4 of culture. All cell nuclei are stained with DAPI (blue). Scale bar = 200 μm.

**Figure 4:**
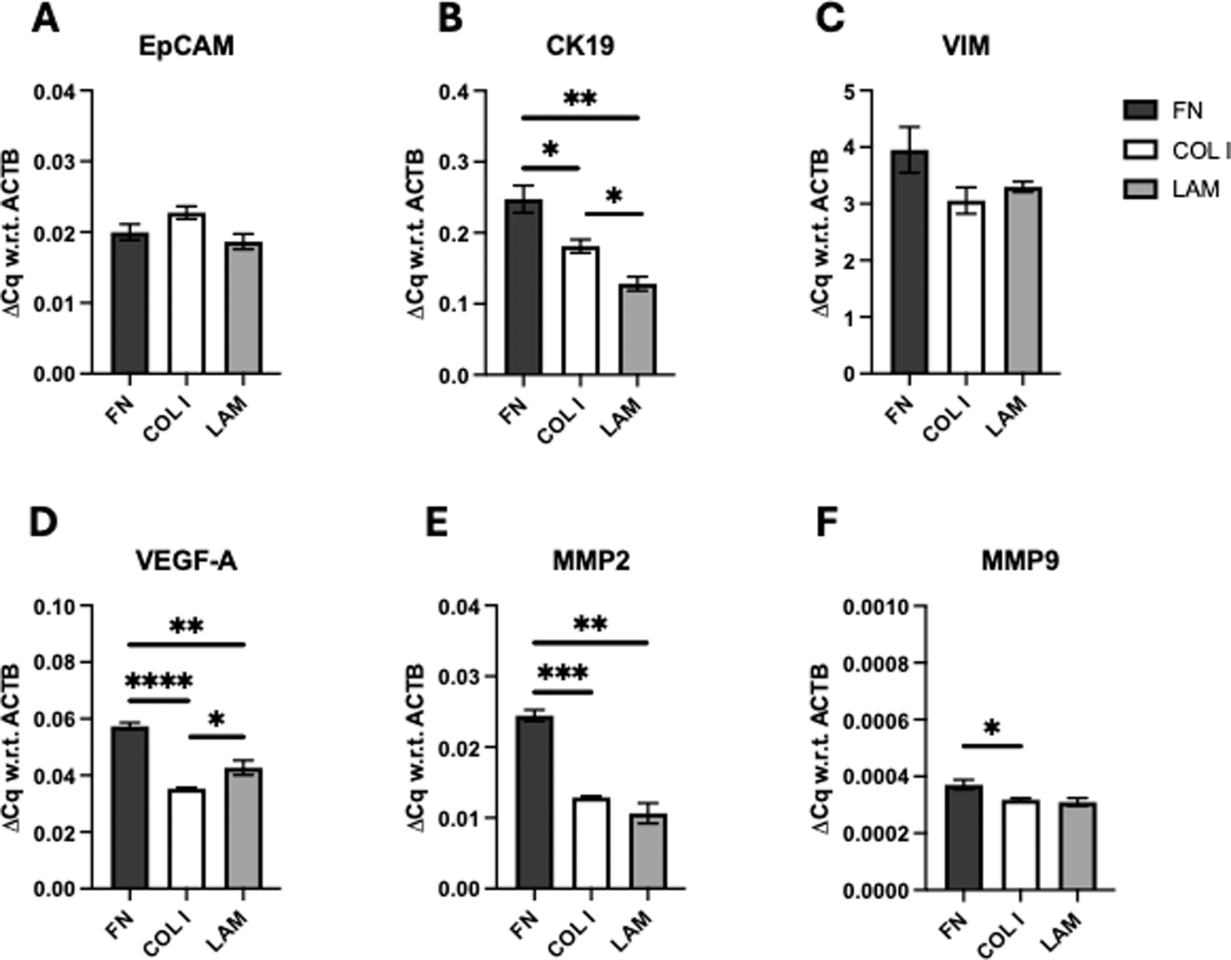
Quantitative analysis of mRNA expressions of PANC-1 pancreatic cancer cells in the single scaffold cancer model for all ECM protein coatings under study at week 4 of culture for (A) epithelial cell adhesion molecule (EpCAM), (B) cytokeratin-19 (CK19), (C) vimentin (VIM), (D) vascular endothelial growth factor-α (VEGFA), MMPs (E) 2 (MMP2) and (F) 9 (MMP9) at week 4 of culture. Fold change (ΔCq) is normalized with respect to (w.r.t) β-actin (ACTB) as the housekeeping gene. An unpaired *t* test was performed, and error bars represent the standard error of the mean (SEM).

**Figure 5:**
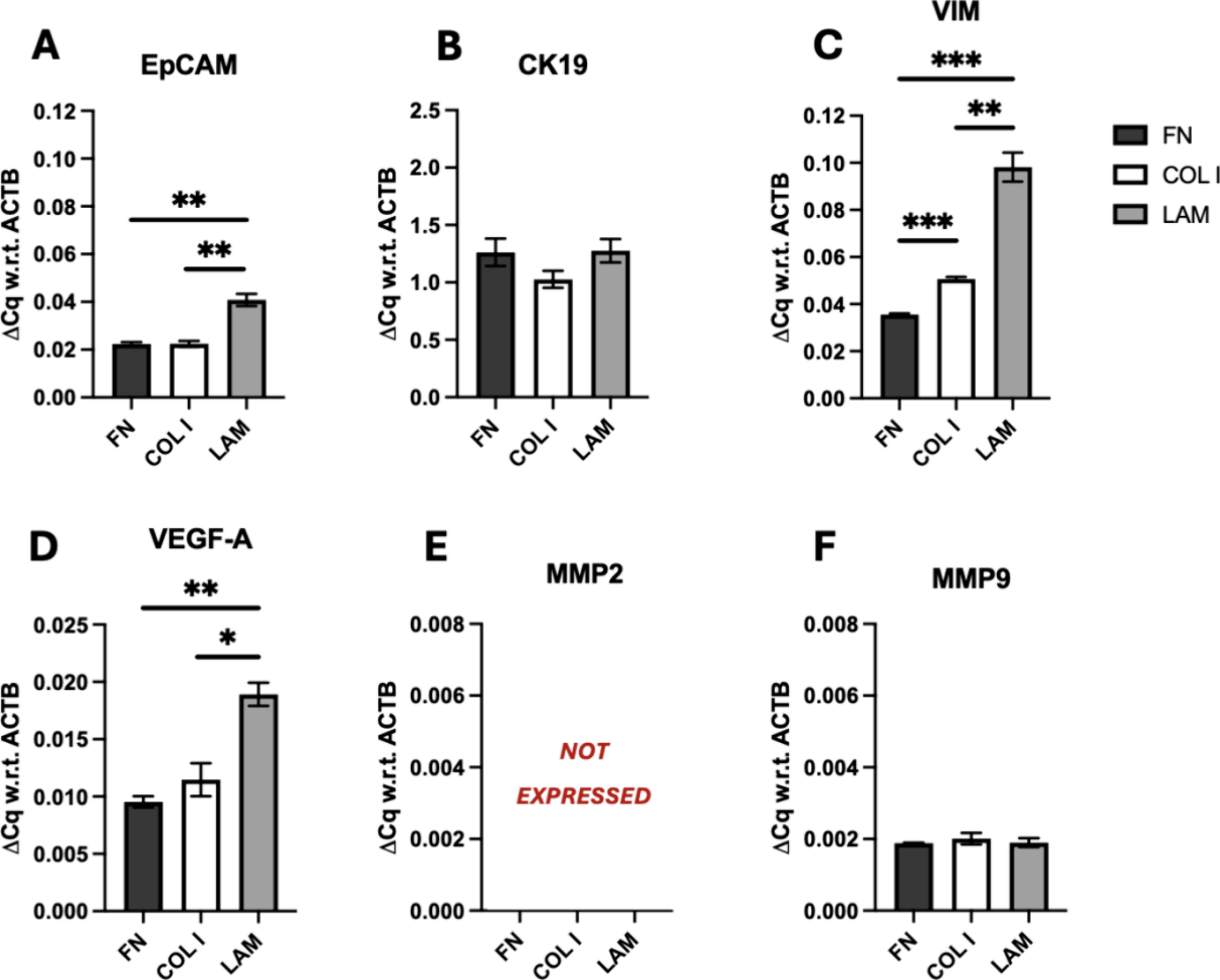
Quantitative analysis of mRNA expressions of BxPC-3 pancreatic cancer cells in the single scaffold stroma model for all ECM protein coatings under study at week 4 of culture for (A) epithelial cell adhesion molecule (EpCAM), (B) cytokeratin-19 (CK19), (C) vimentin (VIM), (D) vascular endothelial growth factor-α (VEGFA), MMPs (E) 2 (MMP2) and (F) 9 (MMP9) at week 4 of culture. Fold change (ΔCq) is normalized with respect to (w.r.t) β-actin (ACTB) as the housekeeping gene. An unpaired *t* test was performed, and error bars represent the standard error of the mean (SEM).

We systematically compared the cellular evolution and biomarker profiling of two different pancreatic cancer cell lines, PANC-1 (highly metastatic) and BxPC-3 (non-metastatic), seeded in single scaffolds coated with three different ECM proteins, i.e., FN, COL I, or LAM (Figure 1). Our results revealed that PANC-1 cells show preference toward FN-coated scaffolds, whereas BxPC-3 cells show preference toward LAM-coated scaffolds in terms of (i) cellular proliferation/denser spatial organization (Figure 2), (ii) ECM protein deposition (Figure 3), and (iii) expression of key biomarkers at the mRNA level, linked to a more aggressive/invasive cancer cell phenotype (Figures 4 and 5).

More specifically, as observed from the viability images (Figure 2 and Supporting Information Figure S1A,B), both PANC-1 and BxPC-3 cancer cell lines exhibited high viability for all three ECM protein coatings under study. However, FN and LAM coatings resulted in significantly more densely populated scaffolds for both cell lines as compared with COL I-coated scaffolds (Figure 2). It should be stated that adhesion was good for all protein coatings under study (no differences in cell egression post-treatment were observed). Furthermore, the density of individual cell clusters was similar, suggesting that most likely, the observed differences in Figure 2 are attributed to cell proliferation in the respective scaffolds. Analysis of the deposition of COL I by both cancer cell lines (Figure 3) revealed interesting trends. More specifically, PANC-1 cancer cells secreted COL I mainly on FN-coated scaffolds and a limited amount on LAM-coated scaffolds, while BxPC-3 cancer cells secreted significant COL I amounts on both FN- and LAM-coated scaffolds. In contrast, no COL I deposition was observed on COL I-coated scaffolds by either of the cancer cell lines.

FN and LAM are basement membrane proteins and thus can exhibit higher affinity for integrin binding compared to non-basement membrane proteins (i.e., COL I). This is known to facilitate cellular attachment and cell growth^105^ and therefore can explain the increased cellular density observed in FN- and LAM-coated scaffolds in this study. There are very limited studies in the literature comparing the pancreatic cancer cell evolution *in vitro*, as affected by different ECM proteins, and none in a similar system to our scaffold. For example, Usman et al. reported differences in cell proliferation for PANC-1 and MiaPaCa-2 pancreatic cell lines in 2D-coated plates, with MiaPaCa-2 showing higher growth in the presence of FN or vitronectin as compared to LN and COL I and PANC-1 cells showing higher growth in the presence of FN, vitronectin, or COL I, as compared to LN. However, when moving to 3D models (PEG hydrogels for MiaPaCa-2), no substantial differences in the cell growth were observed for different ECM proteins.^16^ Aligning with our observations on cell viability in our 3D PU scaffolds, Vaquero et al.^106^ reported increased survival of MiaPaCa-2 and PANC-1 pancreatic cancer cells in the presence of FN and LAM in 2D-coated plates when compared to COL I. More specifically, they reported decreased mitochondrial dysfunction, resulting in reduced necrosis and inhibition of caspase activity, which decreased apoptotic DNA fragmentation as compared to collagen I-coated plates.

Overall, our observations along with the limited existing literature show that (i) there is a clear association of the pancreatic cancer cell behavior to the presence of specific ECM proteins in an *in vitro* system and (ii) this association is also affected by the type of *in vitro* system, i.e., 2D-coated plates vs 3D hydrogels vs 3D scaffolds and the cell line. This points to the necessity of performing comparative studies on cell responses in the presence of ECM within a specific *in vitro* model, as further to the ECM proteins themselves, the characteristics of the model, e.g., the topology, internal structure, or mechanical properties of the model, can affect the cell response.

Further to protein level analysis with imaging, as discussed above, we performed mRNA level analysis of key biomarkers linked to cell aggressiveness/invasiveness (see Section 2.7), i.e., EpCAM, CK19, VIM, MMPs (MMP2 and MMP9), and VEGFA.

As can be seen in Figure 4, PANC-1 cells overexpressed MMP2, MMP9, VEGFA, and CK19 in FN-coated scaffolds, as compared to COL I- and LAM-coated scaffolds. As also mentioned in Section 2.7, MMP2 and MMP9 are linked to PDAC invasion and metastasis, and in patients, they have been associated with poor survival and treatment resistance.^3,94,98−100,104^ CK19 is also expressed in PDAC tissue.^96,101,102^ It is an epithelial marker, also aligned in the literature with the presence of cancer stem cells and increased cancer aggressiveness for pancreatic and hepatocellular carcinomas,^107,108^ while VEGFA is associated with PDAC growth and it is upregulated in the process of angiogenesis.^94,95,97^ We observed no differences in the expression of the epithelial marker EpCAM (which, like CK19, is also associated with the presence of cancer stem cells in the literature^92,93,103^) or the mesenchymal marker VIM^73,91,98,101^ for the scaffold protein coatings under study, suggesting that the mesenchymal phenotype of PANC-1 was unaffected by the protein coatings (Figure 4). Overall, we observed that in our PU scaffolds, FN particularly promotes the upregulation of markers associated with increased PANC-1 aggressiveness/invasiveness.

For BxPC-3 PDAC cells, as can be seen in Figure 5, we observed an upregulation of EpCAM, VEGFA, and VIM on LAM-coated scaffolds as compared to FN- and COL I-coated scaffolds, suggesting that in our 3D scaffolds, LAM promotes aggressiveness/invasiveness and the mesenchymal footprint of BXPC-3 (Figure 5).

In the literature, especially for clinical samples, as mentioned before, both FN and LN are key ECM proteins of the PDAC tissue microenvironment, and they have been associated with the promotion of PDAC aggressiveness and metastatic potential.^13,15,16,109−113^ For example, Hu et al. analyzed 138 PDAC patient samples and detected FN in the tissue of most samples.^114^ The presence of FN was associated with a larger tumor and more advanced disease. Chen et al. analyzed the tissue of 37 pancreatic cancer patients and showed that overexpression of LAM is associated with a more aggressive disease, i.e., fast progression and poor patient prognosis.^12^ However, to the best of our knowledge, there is no *in vitro* paper to date comparing the PDAC expression of the biomarkers we studied (nor others with similar function) in the presence of different ECM proteins in either 2D or 3D systems. Overall, with our data, we show how the phenotype of PDAC cells can be altered in PU scaffolds by changing the protein coating of the scaffold. We clearly show that the proteins stimulating an aggressive or invasive phenotype are cell-line-dependent. Our observations are of importance as simply via altering the ECM coating of the scaffolds, different cell phenotypes can be created (for the same cell line), providing a high throughput system for studies on the progression of the disease, including treatment screening.

### 3.2 3D Stroma Models

**Figure 6:**
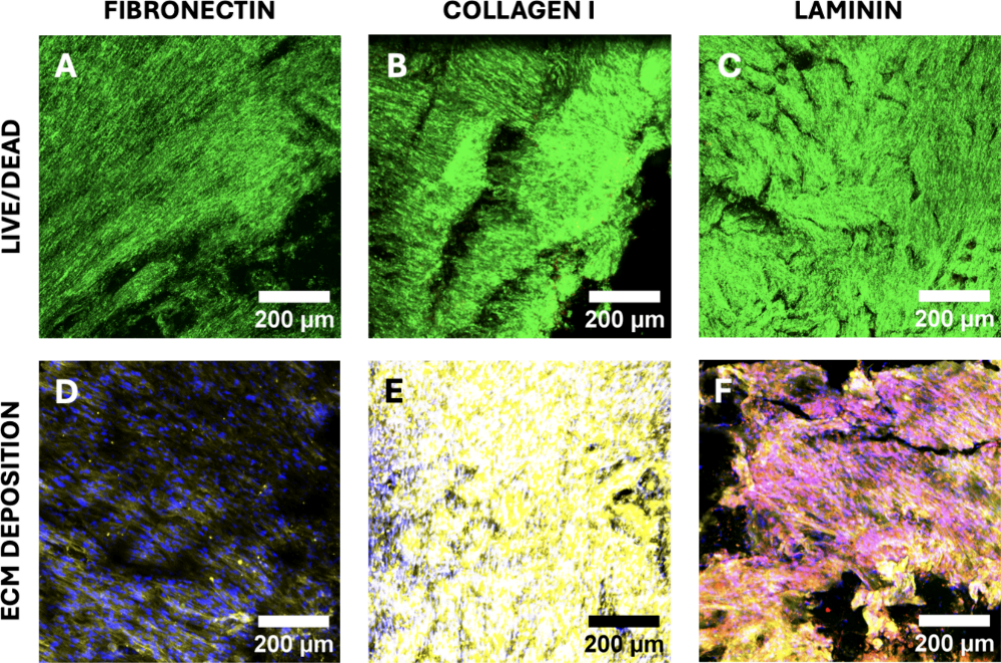
Representative CLSM images of sections of the single scaffold stroma model for all ECM coatings under study at week 4 of culture. Upper panels (A–C) Live–dead staining with green/red areas signifying live/dead cell populations, respectively. Lower panels (D–F): Deposition of ECM proteins (fibronectin (human)—red, collagen I (human)—yellow) with all cell nuclei stained with DAPI (blue). Scale bar = 200 μm.

**Figure 7:**
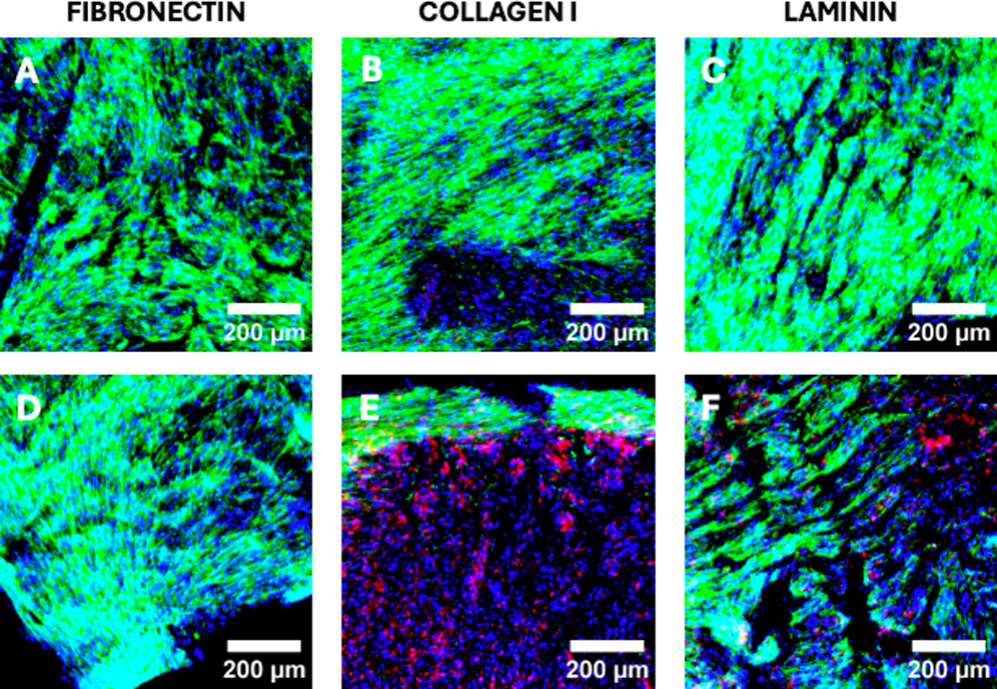
Representative immunofluorescence CLSM images of sections of the single scaffold stroma model for all ECM coatings under study (fibronectin: A+D, collagen I: B+E, laminin: C+F), showing cellular distribution at week 4 of culture. PS-1 stellate cells are imaged in green (αSMA), and HMEC endothelial cells are imaged in red (VWF). All cell nuclei are stained with DAPI (blue). Scale bar = 200 μm.

**Figure 8:**
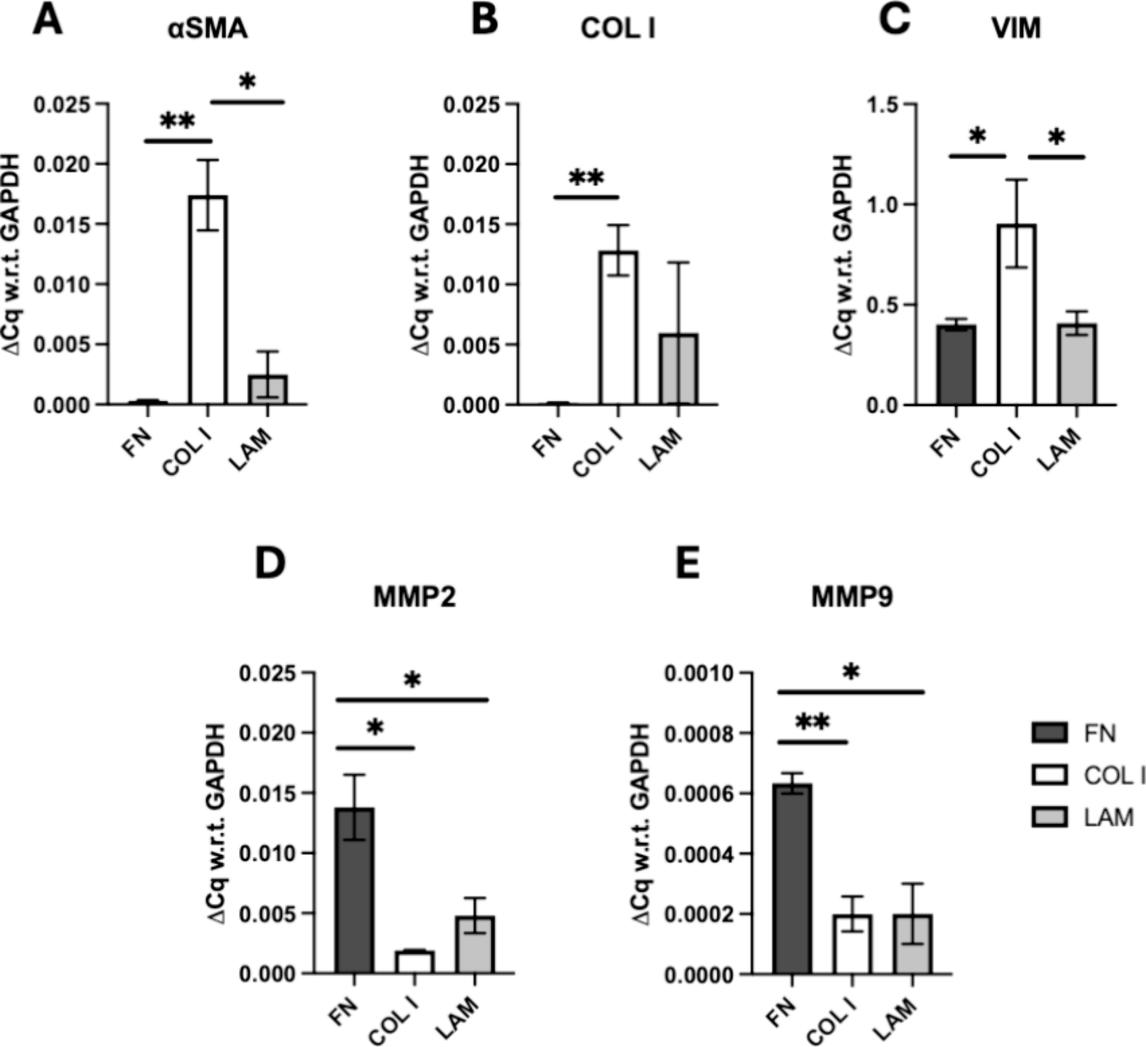
Quantitative analysis of mRNA expressions of stroma cells in the single scaffold stroma models for all ECM coatings under study at week 4 of culture. (A) α-Smooth muscle actin (αSMA), (B) collagen I (COL I), (C) vimentin (VIM), MMPs (D) 2 (MMP2) and (E) 9 (MMP9). Fold change (ΔCq) is normalized with respect to (w.r.t) glyceraldehyde 3-phosphate dehydrogenase (GAPDH) as the housekeeping gene. An unpaired *t* test was performed, and error bars represent the standard error of mean (SEM).

As described above, similar to the cancer cells, we carried out a comparative study to assess the impact of the ECM protein coating on the stroma cells (stellate cells PS-1 and endothelial cells HMEC) and more specifically on (i) the cell viability/spatial aggregation (Figure 6A–C and Supporting Information Figure S1C), (ii) the ECM protein deposition (Figure 6D–F), (iii) the cell-specific distribution (PS-1 vs HMEC) (Figure 7), and (iv) the expression of mRNA level cell-specific markers linked to the functionality of the stroma and to matrix remodeling (Figure 8).

As can be seen in Figure 6 (and viability quantification shown in Supporting Information Figure S1C), the stromal cell population showed a high viability and very high cell density at week 4 of culture for all ECM coatings under study. Comparative analysis of ECM deposition for different ECM scaffold coatings by the stroma cells, i.e., primarily the activated stellate cells, the main role of which is the production of matrix proteins in the PDAC microenvironment,^6,9,17,115,116^ revealed interesting trends. As shown in Figure 6E, stellate cells on COL I-coated PU scaffolds deposited a very high amount of COL I ECM protein, mimicking the desmoplastic reaction associated with PDAC.^35,37^ In contrast, stellate cells within the FN-coated PU scaffolds showed a limited amount of COL I deposition (Figure 6D). Interestingly, in contrast to COL I- and FN-coated scaffolds, LAM-coated PU scaffolds promoted the deposition of both FN and COL I by the stellate cells, with COL I not being as excessively deposited as in COL I-coated scaffolds (Figure 6F). To the best of our knowledge, there is no other study in the literature comparing the *in vitro* matrix deposition of stellate cells or fibroblastic cell lines in the presence of different ECM proteins neither in 2D nor in 3D models. The interesting trends we observe here show the importance and need of conducting such comparative studies in *in vitro* models.

To further assess the presence/spatial density of different cell types (PS-1 and HMEC) within the stroma single scaffold, IF imaging with cell-specific markers was carried out, as previously described (see Section 2.5).^36,37^ As observed in Figure 7, the growth of PS-1 stellate cells was abundant and uniform across all three ECM coatings; however, it is evident that COL I-coated PU scaffolds promoted the growth of HMECs over the FN- and LAM-coated scaffolds. More specifically, large clusters and microneighborhoods of HMECs are present in COL I, with less (and much smaller) clusters present in LAM, and no such clusters present in FN-coated scaffolds. The exact ratio of endothelial/stroma cells shows high heterogeneity (varying from 0% endothelial cells to 70% endothelial cells even for the same coating). In general, the dominant cell type is the activated stellate cells with COL I, promoting the highest microneighborhoods of endothelial cells. Although not extensive, there are limited studies, wherein the effects of ECM proteins on endothelial cell attachment, growth, and proliferation have been studied, however, in 2D *in vitro* models rather than 3D and for a much shorter culturing period. In contrast to our data, most of these studies have suggested that endothelial cells’ growth and proliferation were better supported by FN or LAM coating in 2D plates over other ECM proteins like COL I, Tenascin-C, and Matrigel.^117−119^ It has also been documented in several studies that the benefits of FN on endothelial cell growth in a 2D environment are limited to a short culture period (4 days), post which cellular detachment has been observed.^117−119^ In contrast, we consistently observed HMEC growth and viability for up to 5 weeks within our COL I- and LAM-coated PU scaffolds. In addition, the origin of the cells also plays a role in endothelial cells’ response to the ECM protein within *in vitro* culture conditions.^120,121^ For example, Young et al. investigated the cell attachment of porcine aortic and valve endothelial cells on an FN-coated or a COL I-coated microfluidic device.^122^ They observed that valve endothelial cells showed better spreading over FN, while aortic endothelial cells showed good distribution on COL I. Overall, such variations in findings reported in the literature show that cells can respond very differently to the same protein depending on the *in vitro* model and their origin.

Further elucidation of the cell–cell and cell–ECM interactions in our stroma single scaffolds was carried out through mRNA expression analysis of various markers via q-PCR (see also Section 2.7). As evident in Figure 8, markers associated with activated stellate cells and desmoplasia (α SMA, vimentin, and collagen I) were significantly upregulated within COL I-coated PU scaffolds and moderately upregulated in LAM-coated scaffolds as compared to FN-coated scaffolds, in alignment with the changes on matrix deposition by the stellate cells in the respective scaffold coatings (Figure 6). Although, to the best of our knowledge, there is no reported study comparing such biomarker expression by PS-1 and HMEC cells in a 3D model for different protein coatings, the role of COL I in promoting a 3D adhesive structure formation has been reported for both pancreatic stellate cells and hepatic stellate cells and justifies our observations.^123,124^ More specifically, Yang et al., in an effort to study liver fibrosis, used a modified Boyden chamber coated with COL I (diseased tissue replication) and COL IV (healthy tissue replication). Similarly, to our observation, they reported an upregulation of COL I and MMP2 expression for both rat and human primary hepatic stellate cells in the presence of COL I and FN proteins in comparison to Col IV, Matrigel, and 2D cultures.^124^ Also, in an interesting study that supports our observations, Lombardi et al. studied the effects of aqueous collagen solution on NIH 3T3 murine fibroblast cells in 2D by adding COL I solution in a dose-dependent manner and observed them over 48 h.^125^ They reported the increased expression of collagen I, III, and αSMA by the fibroblasts in the presence of liquid collagen. In terms of metalloproteinase expression (MMP2 and MMP9), we observed a significant upregulation of those on FN-coated scaffolds in comparison to COL I- and LAM-coated scaffolds at week 4 of culture. Aziz-Seible has reported that in the presence of externally added cellular FN, MMP2 secretion was upregulated by hepatocytes,^126^ while Modol et al. had linked externally added FN peptides to increased MMP9 secretion by monocytes.^127^ However, to the best of our knowledge, there are no reported studies to date that have looked at the direct effects of FN or other ECM proteins on MMP expression by PS-1 cells in vitro.

Overall, we show that by varying the ECM coating of our PU scaffolds, we can trigger the PS-1 cells to create different fibrotic/desmoplastic environments in terms of ECM composition and density, as well as in terms of expression of biomarkers associated with invasion. Having such an *in vitro* tool to recreate variations of fibrosis/desmoplasia can help mechanistic studies on the PDAC stroma, including more accurate design and testing of antifibrotic therapies, which show promise in the treatment of PDAC.^128^

### 3.3 Dual Multicellular (Stroma + Cancer) Scaffold Model

We employed our dual scaffold multicellular model (Figure 1),^36,37^ seeded it with both cancer and stroma cells within their, respectively, selected protein coatings, and carried out a comparative study to investigate potential changes in biomarkers linked to aggressiveness/invasiveness as a result of spatial multicellularity. As described in Section 2.3.2, our dual scaffold is composed of a PU core (home of cancer cells) surrounded by an external PU ring (home of stroma cells). This zonal configuration allows spatial modeling of the PDAC *in vivo* microenvironment.^36,37^ Based on our findings from our single scaffold studies (for both the cancer and the stroma), the following protein coatings were chosen: (i) COL I was selected as coating for the external ring, as COL I coating showed a denser fibrosis/desmoplasia (which is a hallmark of the PDAC microenvironment)^2,4,41,129^ in the single scaffold stroma model, along with denser/higher endothelial cell populations, and (ii) FN was selected as protein coating of the cancer core for PANC-1 cells and LAM for BxPC-3 cells, as those coatings led to higher expression of biomarkers linked to a more aggressive/invasive cell phenotype for those cell lines in the single scaffold cancer models (see Sections 3.1 and 3.2).

**Figure 9:**
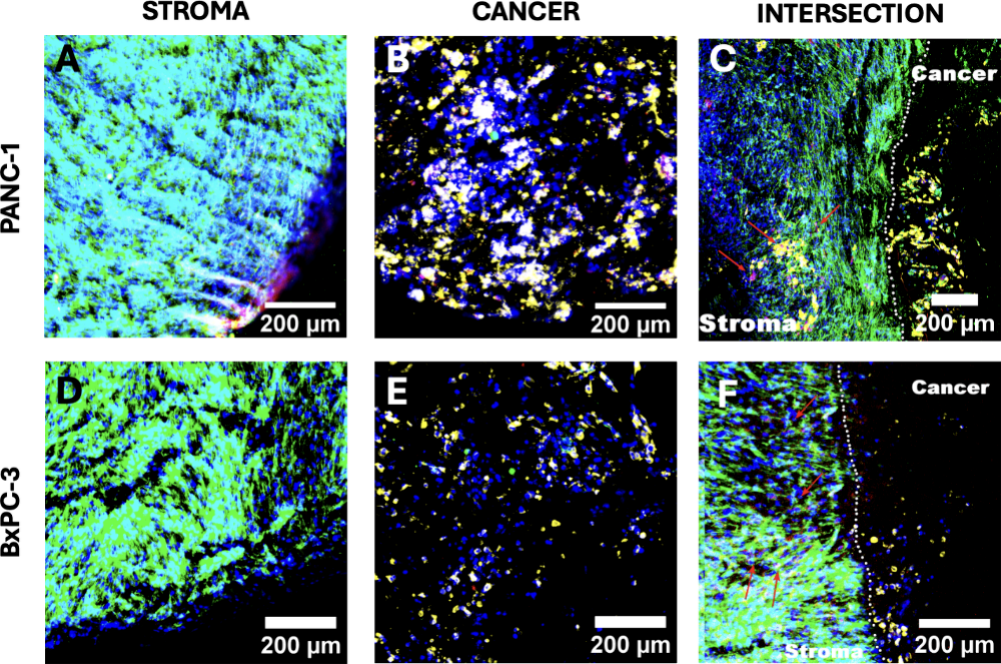
Representative immunofluorescence CLSM images of sections of the dual multicellular scaffold model at week 4 of culture. Upper panel (A–C): PANC-1 cancer cells are seeded in a fibronectin (FN)-coated inner core, and stroma cells (HMEC and PS-1) are seeded in a collagen I (COL I)-coated outer stroma. Lower panel (D–F): BxPC-3 cancer cells are seeded in a laminin (LAM)-coated inner core, and stroma cells (HMEC and PS-1) are seeded in a collagen I (COL I)-coated outer stroma. PANC-1 and BxPC-3 pancreatic cancer cells are imaged in yellow, PS-1 stellate cells are imaged in green, and HMEC endothelial cells are in red. All cell nuclei are stained with DAPI (blue). Scale bar = 200 μm.

Immunofluorescence imaging of cell-specific markers within our 3D *in vitro* dual scaffold model of pancreatic cancer (Figure 9) reveals a densely populated cellular environment, particularly within the outer ring, showing a good spatial mimicry of the PDAC desmoplastic reaction, wherein cancer masses are typically surrounded by a dense fibrotic layer.^2,4,19,33,41,129,130^ As can be seen in Figure 9, in our spatial models, the cancer and stroma cell niches establish distinct microenvironments; however, they are also interspersed but in a cancer cell-line-dependent way. More specifically, as can be seen in Figure 9C, for the metastatic cell line PANC-1, cancer cells have infiltrated the stroma. At the same time, both activated stellate cells and endothelial cells have invaded within the cancer compartment, forming microneighborhoods within the cancer mass and at substantial distances (min distance of ∼488 μm and max distance of ∼1382 μm) from the cancer intersection. This clearly indicates the communication and migration of the cells between the two compartments. Additionally, the presence of endothelial cells moving into the cancer compartment suggests communication and migration between the tumor and its surrounding stroma, which could be critical for promotion of tumor progression and/or vascularization.^131−133^ Interestingly, no such migration of cells for neither the cancer nor the stroma compartments is observed for the nonmetastatic BxPC-3 cell line (Figure 9F). There are very limited spatial 3D models of PDAC,^80,134,135^ from which the model of Pape et al. spatially observes invasion. More specifically, Pape et al. developed a matrix-rich dual hydrogel consisting of a pancreatic cancer (MiaPaCa-2 cell line) core surrounded by an acellular matrix-rich periphery to represent the ECM of the stroma. They observed migration/invasion of the cancer mass toward the matrix-rich periphery.^134^ Our observations (Figure 9) highlight the capability of our model to provide a basis for *in vitro* migration and invasion studies. At the same time, the differences observed for the two pancreatic cancer cell lines are in line with their cellular nature, i.e., metastatic vs nonmetastatic, and show the capability of our 3D system to maintain the cancer cell phenotypes long-term (4 weeks in culture).

**Figure 10:**
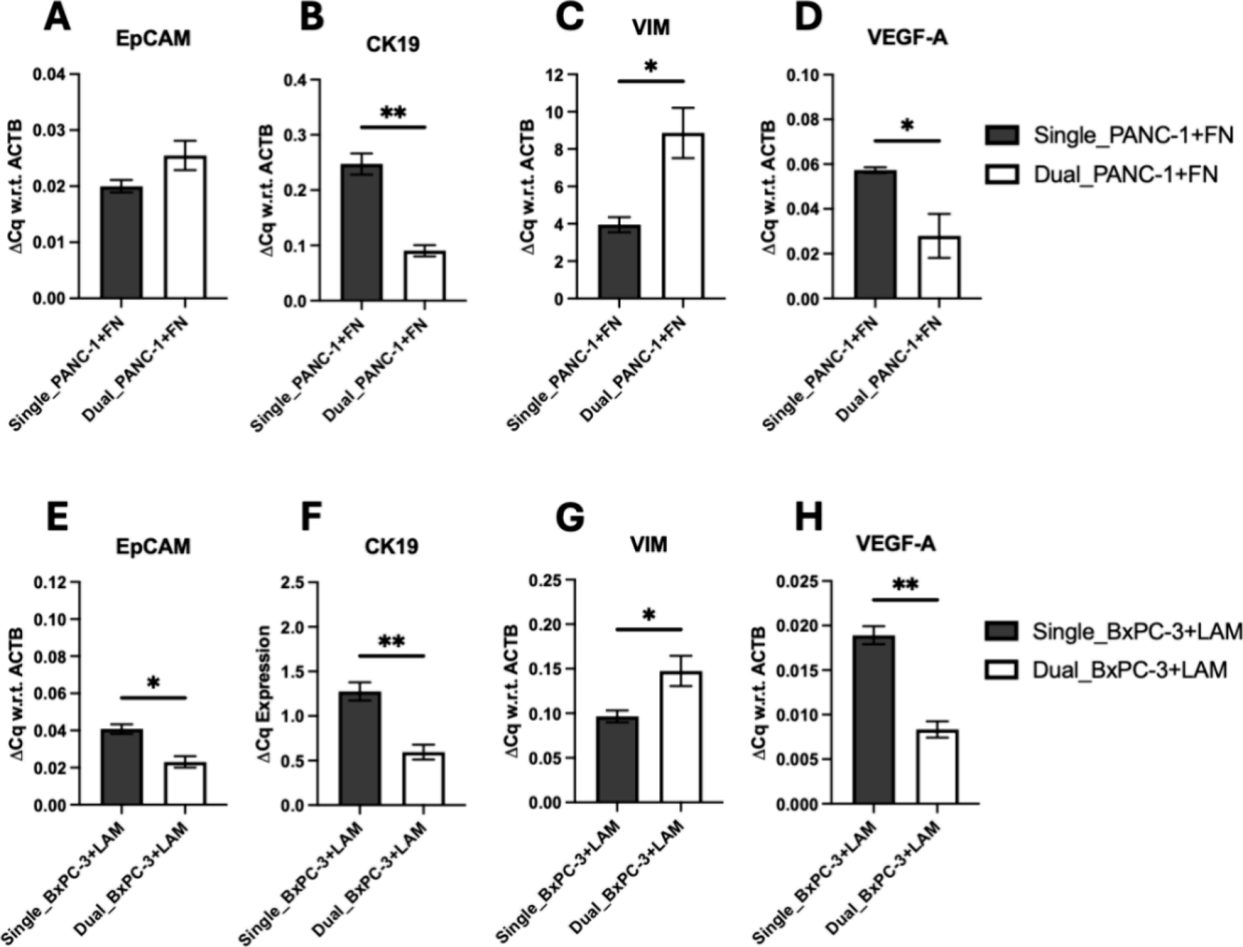
Quantitative comparison of mRNA expressions of pancreatic cancer cells (PANC-1: A,B,C,D; BxPC-3:E,F,G,H) extracted from the core of the dual multicellular scaffolds as compared to the single scaffold cancer models at week 4 of culture for epithelial cell adhesion molecule (EpCAM), cytokeratin-19 (CK19), vimentin (VIM), and vascular endothelial growth factor-α. The following coatings are used: Fibronectin coating for PANC-1 cells and laminin coating for BxPC-3 cells (in both dual and single scaffold configurations). Fold change (ΔCq) is normalized with respect to (w.r.t) ACTB as the housekeeping gene. An unpaired *t* test was performed, and error bars represent the standard error of the mean (SEM).

**Figure 11:**
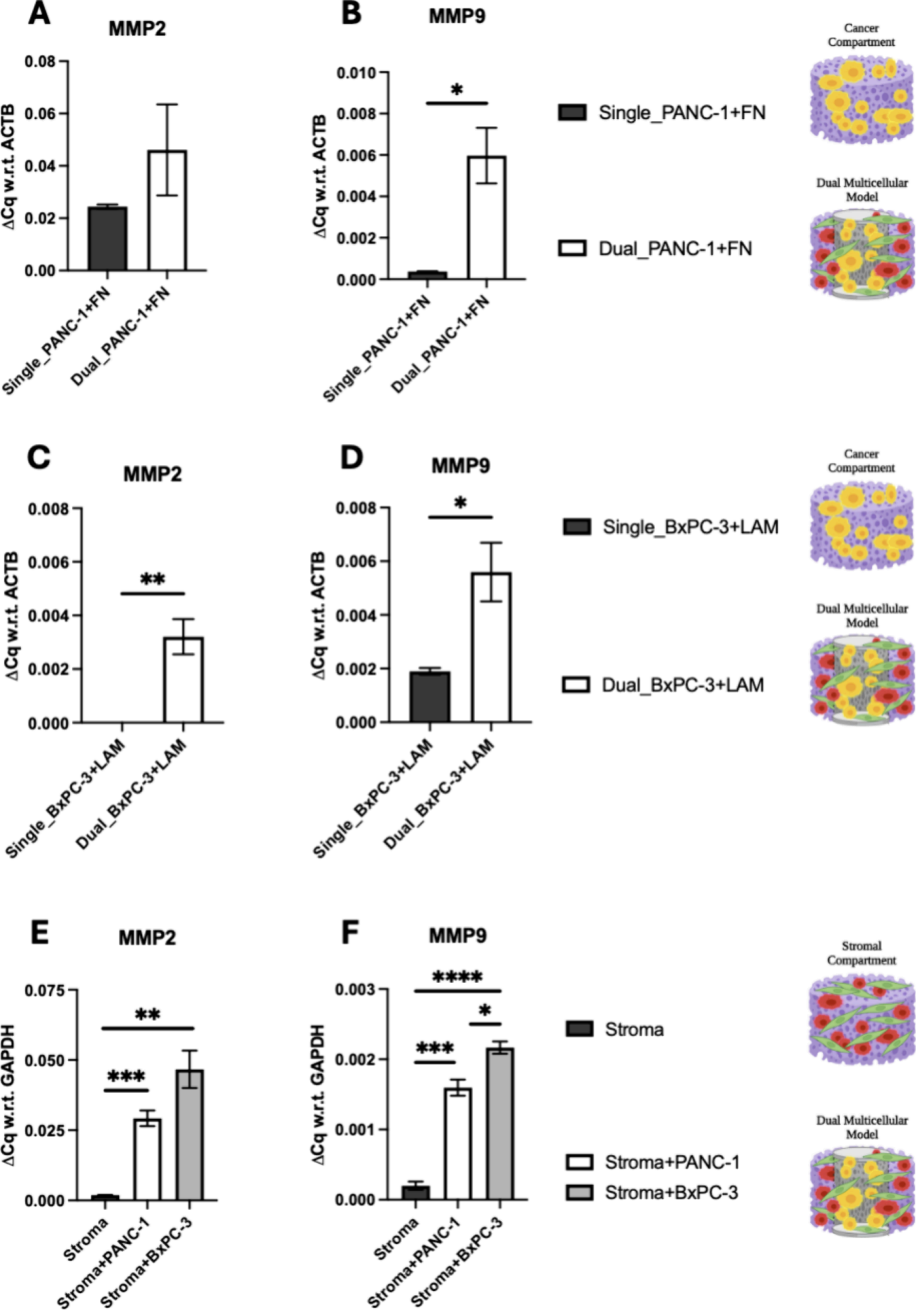
Quantitative comparison of mRNA expressions for MMPs 2 (MMP2) and 9 (MMP9) for pancreatic cancer cells (PANC-1: A–B, BxPC-3: C–D) or stroma cells (E,F) extracted from the dual scaffolds as compared to the cancer single scaffolds at week 4 of culture. The following coatings are used: Fibronectin coating for PANC-1 cells, laminin coating for BxPC-3 cells and collagen I coating for stroma cells (in both dual and single scaffold configurations). Fold change (ΔCq) is normalized with respect to (w.r.t) ACTB and GAPDH as housekeeping genes for cancer and stromal compartments, respectively. An unpaired *t* test was performed, and error bars represent the standard error of the mean (SEM).

On an mRNA expression level, as can be seen in Figure 10, we observed downregulation of epithelial markers (EpCAM and CK19) and upregulation of the mesenchymal marker VIM for both PANC-1 and BxPC-3 cancer cell lines in the cancer core in the multicellular dual scaffold model, as compared to their respective single scaffold cancer model counterparts. However, a significant decrease in VEGFA was detected for PANC-1 and BxPC-3. Furthermore, we generally see an upregulation of the expression of MMP2 and MMP9 for both the cancer and stroma compartments and for both cancer cell lines, as compared to their monocellular cancer-only or stroma-only single scaffolds (Figure 11).

The changes observed for the above biomarkers further show the presence of signaling between cancer and stroma cells in the dual scaffold. As previously mentioned, MMPs are crucial for ECM remodeling and have been strongly linked to cancer progression and metastasis. Their elevated levels in our dual scaffold model suggest an enhanced invasive potential and increased treatment resistance potential in PDAC cells.^31,99,100,104^ This increased expression of MMPs in the dual scaffold model corroborates previous studies that have demonstrated the stroma component’s role in promoting upregulation of MMPs.^31,99,100,104,136,137^ For example, Xu et al. performed immunohistochemical analysis in 103 pancreatic cancer patient specimens and found that the expression of MMP9 was increased in all patient tissues compared to healthy pancreatic tissues, and MMP9 was overexpressed in the stroma areas of the patients’ tissue.^104^

The downregulation of epithelial markers (EpCAM, CK19) with the contemporary upregulation of the mesenchymal marker VIM (Figure 10) shows a shift toward a mesenchymal phenotype, which is associated with epithelial-to-mesenchymal transition,^73,92,93,96,98,101−103^ increased metastatic and resistance potential, and clearly shows the increased cell aggressiveness/invasiveness that the spatial multicellularity introduces in our system. Kikuta et al.^101^ observed an overall upregulation of mesenchymal markers (VIM and SNAI-1) and downregulation of epithelial markers (E-cadherin and CK19) when co-culturing pancreatic cancer cells (PANC-1 and SUIT-2) with PSCs in a spheroid model as compared to monocellular cancer-only spheroids, indicating reduced cell adhesion and increased invasiveness. Similarly, Pape et al., reported upregulation of biomarkers linked to epithelial-to-mesenchymal transition and treatment resistance in their hydrogel-based spatial PDAC tumoroid model.^134^ Monteiro et al. also reported an upregulation of markers related to invasiveness, EMT, and drug resistance (e.g., TGF-β) in a GelMA-Hyaluronan hydrogel-based spatial model consisting of a PANC-1 core surrounded by CAFs, as compared to monocellular hydrogels.^80^ We observed downregulation of VEGFA in our multicellular models for both PANC-1 and BxPC-3. Although it is well reported that VEGFA is upregulated by cancer as a result of the fibroblastic fibrosis,^94,95,97^ there is also literature, suggesting that endothelial cells can secrete soluble factors that downregulate the VEGFA expression by cancer cells.^97,138^

Overall, there is very limited literature on spatially advanced models of PDAC. For example, Monteiro et al. developed a “cancer-on-a-bead” spatial model using PANC-1 cancer cells to create a cancer core surrounded by CAFs as the stroma and encapsulated both compartments in GelMA-Hyaluronan hydrogels.^80^ The study found that those heterotypic cancer-on-a-bead models exhibited a higher resistance to gemcitabine treatment and higher expression of resistance-related markers (TGF-β1 and CXCL12) compared to their monoculture cancer-only counterparts. Also, as previously mentioned, Pape et al. developed a 3D spatial pancreatic tumoroid model (cancer core surrounded by complex matrix), wherein it was demonstrated that increased matrix complexity resulted in increased invasiveness and chemoresistance of the cancer cells.^134^ This limited literature available in different 3D models to our scaffolds highlights similarities to our findings with respect to changes in the cancer cell invasiveness/aggressiveness as compared to monocellular models and indicates the importance of considering spatial complexity when designing *in vitro* cancer models.

Our multicellular platform provides a unique system, wherein future studies on the impact of desmoplasia on the progression of pancreatic cancer can be conducted. More specifically, by altering the coating of the periphery (stroma compartment), different desmoplastic environments can be recreated *in vitro* (in terms of type and level of matrix deposition) to surround a specific cancer core. At the same time, for a given desmoplastic composition of the stroma, different cancer cores can be recreated for the same cancer cells to be used as a basis for crosstalk studies between the cancer and the stroma populations of the tumor tissue microenvironment.

## 4 Conclusions

Using the PU-based PDAC models that we previously developed,^5,27,35−38^ we performed a systematic study on how different ECM protein coatings (FN, LAM, COL I) of our PU scaffolds impact the long-term (4 weeks) evolution, namely, the viability, ECM deposition, and expression of biomarkers linked to cell aggressiveness/invasiveness in scaffolds containing only cancer or only stroma cells (activated stellate cells and endothelial cells). Thereafter, to investigate potential further changes in those biomarkers as a result of cancer–stroma interactions, we studied their upregulation in our dual/zonal scaffolds, consisting of a cancer core and a stroma periphery, for selected scaffold protein coatings.

In our single scaffold models, we observed that the protein coating affected the cancer cell progression, matrix deposition, and biomarker upregulation in a cell-line-dependent manner. More specifically, metastatic PANC-1 pancreatic cancer cells developed a more aggressive phenotype for FN-coated scaffolds while the nonmetastatic BxPC-3 cells for LAM-coated scaffolds. We can therefore create biomaterial-based environments, wherein the same cell type can develop different levels of invasiveness/aggressiveness. For the single scaffold stroma models, we showed that although all ECM proteins supported very high cell density formation, the functionality of stellate cells, as well as the presence of endothelial cells, was impacted by the protein coating. More specifically, different levels of fibrosis/desmoplasia, in terms of ECM composition and quantity, were generated for different ECM coatings. COL I scaffold coating led to the densest desmoplasia and to the highest COL I deposition by the stellate cells, as well as to the presence of the largest microneighborhoods of endothelial cells, while FN coating led to the lowest/scarcest desmoplasia. LAM supported the secretion and deposition of large amounts of FN further to collagen by the stellate cells.

For selected protein coatings, when studying the evolution of cancer and stroma cells in our dual/zonal model, consisting of a cancer core surrounded by a stroma periphery that spatially mimics the PDAC tissue microenvironment, we show further upregulation of biomarkers linked to cell aggressiveness/invasiveness by both the cancer and stroma cells as compared to monocellular models (cancer only or stroma only scaffolds). Furthermore, we show infiltration of the stroma by the tumor, as well as migration of both stellate and endothelial cells to the cancer core for metastatic PANC-1 cells but no such cell movement in models consisting of nonmetastatic BxPC-3 cells.

Collectively, our study advances the understanding of how different ECM proteins impact the long-term cell evolution, and our findings show that within our bioengineered models, we can stimulate the cells of the PDAC microenvironment to develop different levels of aggressiveness/invasiveness, as well as different levels of fibrosis. Furthermore, we highlight the importance of considering spatial complexity to map cell invasion. These insights contribute to the broader scientific effort to design more biomimetic models to study and elucidate the TME’s role in cancer progression *in vitro*.

Future research should focus on understanding the specific signaling pathways and interactions between cancer cells and stroma components as it could reveal new therapeutic targets for pancreatic cancer. Furthermore, similar comparative studies in other 3D models are needed, especially as the majority of available literature does not modulate the ECM composition within a specific 3D model, and that is important as the model configuration can alter the cell response. Finally, our platform can be used as a basis for studying other highly fibrotic cancers, as well beyond pancreatic cancer (such as liver cancer).

## Supporting Information Available

Primary and secondary antibodies used for immunofluorescence staining; list of primer pairs used for assessment of gene expression via qPCR in our single and dual scaffold PDAC models; and viability quantification (PDF)
