# Supplementary material for: Mapping Tumor–Stroma–ECM Interactions in Spatially Advanced 3D Models of Pancreatic Cancer
Source: ACS Appl Mater Interfaces. 2025 Mar 7;17(11):16708–24. doi: 10.1021/acsami.5c02296 (PMC11931495; doi:10.1021/acsami.5c02296)
Supplement: Supplementary file 1 — am5c02296_si_001.pdf [file am5c02296_si_001.pdf]

Supporting Information

Mapping Tumor-Stroma-ECM Interactions in Spatially Advanced 3D Models of Pancreatic Cancer

Anna-Dimitra Kataki [a], Priyanka G. Gupta [a, b], Umber Cheema [a], Andrew Nisbet [c], Yaohe Wang [d], Hemant M. Kocher [e], Pedro A. Pérez-Mancera [f] and Eirini G. Velliou [a*]

[a] *Centre for 3D models of Health and Disease, Division of Surgery and Interventional Science, University College London, London W1W 7TY, UK*

[b] *School of Life and Health Sciences, Whitelands College, University of Roehampton, London SW15 4JD, UK.*

[c] *Department of Medical Physics and Biomedical Engineering, University College London, London WC1E 6BT, UK.*

[d] *Centre for Cancer Biomarkers and Biotherapeutics, Barts Cancer Institute, Queen Mary University of London, London EC1M 6BQ, UK.*

[e] *Centre for Tumour Biology and Experimental Cancer Medicine, Barts Cancer Institute, Queen Mary University of London, London EC1M 6BQ, UK.*

[f] *Department of Molecular and Clinical Cancer Medicine, University of Liverpool, Liverpool L69 3GE, UK.*

[*]Corresponding author email: e.velliou@ucl.ac.uk

Supplementary Note #1:

The primary and secondary antibodies used for the conduction of immunohistochemistry are summarised in Table S1, below.

__**Table S1:**__ Primary and secondary antibodies used for immunofluorescence staining. All primary antibodies and Dylight secondary antibodies were obtained from Abcam UK. Alexa Fluor secondary antibodies were obtained from Thermo Fisher, UK.

**FIGURES**	**PRIMARY ANTIBODY**	**SECONDARY ANTIBODY**
Figure 9	Mouse anti human anti Pan Cytokeratin (Isotype: Mouse IgG1)	Donkey anti-Mouse Secondary Antibody, DyLight 550
Figures 7, 9	Rabbit anti human anti Von Willebrand Factor (Isotype: Rabbit IgG)	Donkey anti-Rabbit secondary Antibody, DyLight 650
Figures 7, 9	Rabbit anti human anti Alpha Smooth Muscle Actin – AF 488 (Isotype: AF 488 Rabbit IgG)	n/a
Figures 3, 6	Rabbit anti human anti Collagen I (Isotype: Rabbit IgG)	Donkey anti Rabbit- AF555- Secontrary antibody
Figures 3, 6D-F	Rat anti human anti Laminin 2 (Isotype: Rat IgG1)	Donkey Anti Rat AF 488-Secontrary antibody
Figures 3, 6D-F	Mouse anti human anti Fibronectin (Isotype: Mouse IgG1)	Donkey Anti Mouse AF647- Secontary antibody

Supplementary Note #2:

The primer pairs used to performed qPCR are outlined in Table S2 below.

__**Table S2:**__ List of primer pairs used for assessment of gene expression via qPCR in our single and dual scaffold PDAC models.

**GENE SYMBOL**	**FORWARD (5'to 3')**	**REVERSE (5'to 3')**
GAPDH [1]	CGGAGTCAACGGATTTGGTC	TTCCCGTTCTCAGCCTTGAC
ACTB	TTCAACACCCCAGCCATGT	GCCAGTGGTACGGCCAGA
COL I [1]	GAGGGCCAAGACGAAGACATC	CAGATCACGTCATCGCACAAC
MMP2 [1]	CCCACTGCGGTTTTCTCGAAT	CAAAGGGGTATCCATCGCCAT
MMP9 [1]	TAGGGCTCCCGTCCTGCTT	CCACCTCCACTCCTCCCTTTC
VEGFA [1]	AATCGAGACCCTGGTGGACATC	TTGATCCGCATAATCTGCATGG
EpCAM	TTGCTGTTATTGTGGTTGTGGTG	CACCCATCTCCTTTATCTCAGCC
VIM	TCCAAGTTTGCTGACCTCTCTG	CGTTCCAGGGACTCATTGGTTC
CK19	AGCCGGACTGAAGAATTGAACC	TTCCAAGGCAGCTTTCATGCTC

Supplementary Note #3:

We performed quantification of the viability images .i.e., Figures 2 and 6 (based on the ratio of live over total live plus dead areas of our images, as we have previously described[1-4]) for all cells under study. The quantification trends are summarised in Figure S1, and demosnstrate high cell viability across all conditions under study.

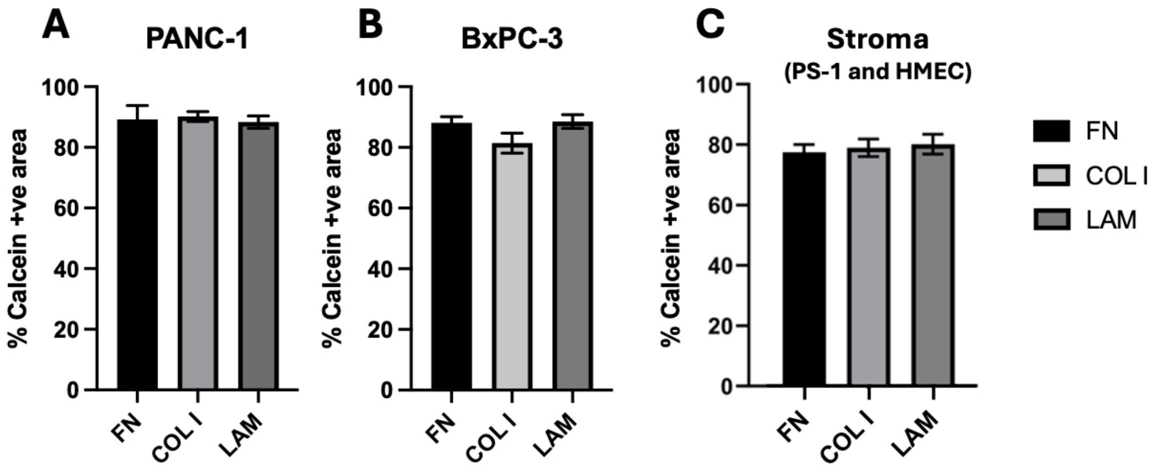

<u>Figure S1:</u> Viability quantification of % Calcein +ve area (live area) of the single scaffold models for all three ECM protein coatings under study for (A) PANC-1 cancer cells (B) BxPC-3 cancer cells and (C) stroma cells at week 4 of culture. Unpaired *t* test was performed, and error bars are representing standard error of mean (SEM).

References:

(1) Gupta, P.; Bermejo-Rodriguez, C.; Kocher, H.; Perez-Mancera, P. A.; Velliou, E. G. Chemotherapy Assessment in Advanced Multicellular 3D Models of Pancreatic Cancer: Unravelling the Importance of Spatiotemporal Mimicry of the Tumor Microenvironment. *Adv Biol (Weinh)* **2024**, *8* (7), e2300580. DOI: 10.1002/adbi.202300580.

(2) Wishart, G.; Gupta, P.; Nisbet, A.; Velliou, E.; Schettino, G. Enhanced effect of X-rays in the presence of a static magnetic field within a 3D pancreatic cancer model. *Br J Radiol* **2023**, *96* (1143), 20220832. DOI: 10.1259/bjr.20220832.

(3) Gupta, P.; Totti, S.; Pérez-Mancera, P. A.; Dyke, E.; Nisbet, A.; Schettino, G.; Webb, R.; Velliou, E. G. Chemoradiotherapy screening in a novel biomimetic polymer based pancreatic cancer model. *RSC Advances* **2019**, *9* (71), 41649-41663. DOI: 10.1039/c9ra09123h.

(4) Totti, S.; Allenby, M. C.; Dos Santos, S. B.; Mantalaris, A.; Velliou, E. G. A 3D bioinspired highly porous polymeric scaffolding system for in vitro simulation of pancreatic ductal adenocarcinoma. *RSC Advances* **2018**, *8* (37), 20928-20940. DOI: 10.1039/c8ra02633e.
